# Predictive factors for 6 *vs* 12 cycles of Folfiri-Bevacizumab in metastatic colorectal cancer

**DOI:** 10.18632/oncotarget.23355

**Published:** 2017-12-17

**Authors:** Vincenzo Formica, Maria Teresa Ionta, Bruno Massidda, Giacomo Vessia, Luigi Maiorino, Rossana Casaretti, Donato Natale, Giuseppe Barberis, Gianfranco Filippelli, Ettore Greco, Livio Blasi, Sergio Mancarella, Anna Russo, Enrico Barbato, Liberato Di Lullo, Mario Roselli

**Affiliations:** ^1^ Department of Systems Medicine, Medical Oncology Unit, Tor Vergata University Hospital, Rome, Italy; ^2^ Medical Oncology II, Azienda Ospedaliero, Universitaria di Cagliari, Cagliari, Italy; ^3^ Oncologia Medica, Ospedale Della Murgia, Altamura, Italy; ^4^ Department of Medical Oncology, Napoli, Italy; ^5^ Istituto Nazionale per lo Studio e la Cura dei Tumori “Fondazione G. Pascale”- IRCCS, Naples, Italy; ^6^ Ospedale Civile San Massimo, Pescara, Italy; ^7^ Oncologia Medica, Ospedale Evangelico Villa Betania, Napoli, Italy; ^8^ Ospedale S. Francesco di Paola, Paola CS, Italy; ^9^ Oncologia Medica, P.O. Lamezia Terme, Italy; ^10^ UOC Oncologia Medica, ARNAS Civico, Palermo, Italy; ^11^ Oncologia Medica, Presidio Ospedaliero S Caterina, Galatina, Italy; ^12^ Oncologia Medica, Policlinico “Paolo Giaccone”, Palermo, Italy; ^13^ Oncologia medica, Ospedale “ Moscati “ Aversa, Aversa, Italy; ^14^ Oncologia Medica, Ospedale F. Veneziale, Isernia, Italy

**Keywords:** metastatic colorectal cancer, irinotecan, fluorouracil, bevacizumab, death pace analysis

## Abstract

Early switching to de-intensified maintenance regimen is still a matter of debate in metastatic colorectal cancer (mCRC).

The MARTHA trial, a S.I.C.O.G. phase III randomized trial, compared FOLOFIRI+bevacizumab (B) for 12 cycles (6 months) followed by B for up to 12 months (FOLFIRI +B*12 arm) *vs* FOLFIRI+B for 6 cycles (3 months) followed by capecitabine+B for 4 cycles followed by B for up to 12 months (FOLFIRI+B*6 arm). Chemotherapy-naïve mCRC patients were randomized, primary endpoint was progression free survival (PFS), with overall survival (OS) as a secondary endpoint. A novel analysis, the Death Pace Analysis (DPA), was performed to identify patients who benefited from a specific treatment.

No PFS difference was seen in 198 enrolled patients (101 in FOLFIRI+B*12, 97 in FOLFIRI+B*6). A non-significant superior OS was observed for FOLFIRI+B*6 (HR 0.74, p 0.098). The DPA demonstrated that 14% of patients were identifiable as FOLFIRI+B*6-benefiting patients. According to a logistic regression analysis including 23 clinicopathological variables, baseline Hb was the only independent predictor of DPA-defined FOLFIRI+B*6-benefit status. Among patients with Hb ≤ 11.1 gr/dL a statistically significant prolonged OS was observed for FOLFIRI+B*6 over FOLFIRI+B*12 (median OS: 20.7 *vs* 12.6 months, respectively, HR 0.54, p 0.048). No survival difference was observed between arms in patients with Hb > 11.1.

mCRC patients with low baseline Hb levels are better treated with FOLFIRI+B*6 first-line strategy. Possible biological explanations for this finding are being investigated.

## INTRODUCTION

Inoperable metastatic colorectal cancer (mCRC) is an incurable disease for which the main objective of systemic treatment is to stabilize or reduce the tumor burden, thus prolonging survival while maintaining quality of life [1].

In the last two decades the therapeutic armamentarium has broadened significantly, and median overall survival has passed from 10 months with the sole use of fluorouracil in 1980s-1990s to the current 32-40 months with the use of several novel biologic and chemotherapeutic agents [2]. Molecular refinement with complete RAS and BRAF gene mutational analysis has proved to be crucial for treatment selection and outcome improvement [3]. Lately, a simple clinical feature, the primary tumour location (right *vs* left colon), has been demonstrated to have a significant impact on prognosis, and perhaps on the efficacy of anti-EGFR agents, in wild type colorectal cancer patients [4]. Possible molecular and microenviromental differences between right-sided and left-sided tumours have been put forth to explain this clinical finding [5].

The focus on safeguarding quality of life over a prolonged time of treatment exposure (up to > 40 months) has increasingly gained attention [6].

A widely adopted approach to guarantee an adequate quality of life and limit cumulative toxicity is the interruption of full dose treatment after six to eight months and the start of maintenance de-intensified treatment with single biologic agents with or without fluorpyrimidine [7–9].

A further reduction of the full treatment period from (three months instead of 6-8 months) may significantly limit mid-term side effects and delay quality of life deterioration.

FOLFIRI+bevacizuamab (B) delivered until disease progression is one of the standard first-line regimens for mCRC patients [10, 11]. The MARTHA (Maintenance and Reduction Chemotherapy With Avastin in Metastatic Colon Cancer) study is a phase III multicenter randomized trial carried out within the S.I.C.O.G. (Southern Italy Cooperative Oncology Group) that compares efficacy and toxicity of the following two treatment strategies: FOLFIRI+B for 12 cycles (six months) followed by maintenance B monotherapy for up to 12 months (FOLFIRI+B*12 arm) *vs* FOLFIRI+B for six cycles (three months) followed by capecitabine+B for three months followed by B monotherapy for up to 12 months (FOLFIRI+B*6 arm). In the present article efficacy and safety data are reported together with a novel analysis, the Death Pace Analysis (DPA) [12, 13], which aims to identify patients more likely to derive survival benefit from a specific treatment. Furthermore, clinicopathological factors that might identify DPA-defined patients were assessed using standard logistic regression analysis. Primary endopoint was progression free survival (PFS), overall survival (OS) was a secondary endpoint together with objective response rate (ORR) and safety.

## RESULTS

### Efficacy

**Figure 1:**
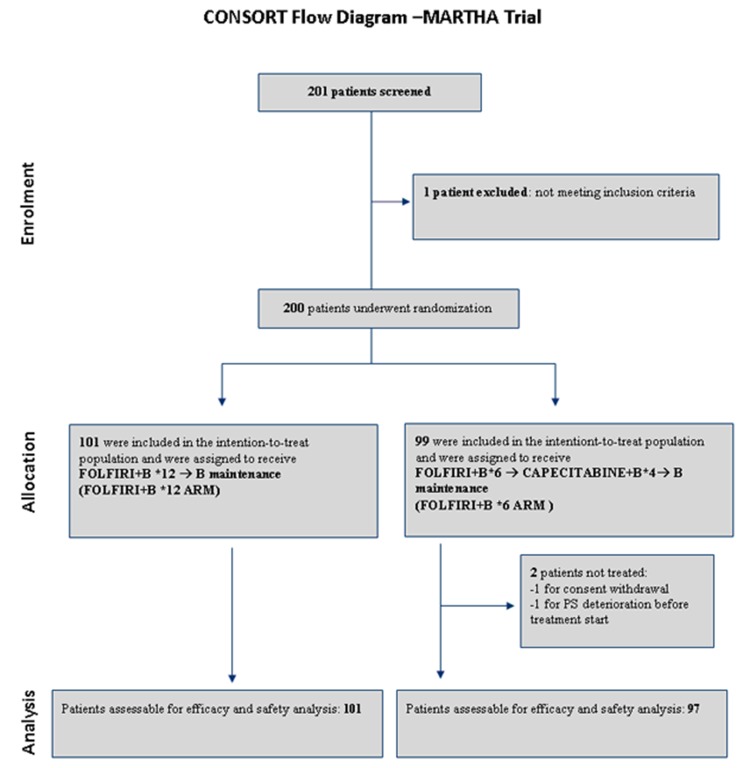
CONSORT diagram of MARTHA trial B: bevacizumab. PS: performance status

From August 2008 to October 2012, 198 patients (101 in FOLFIRI+B*12 arm, 97 in FOLFIRI+B*6 arm) were enrolled and randomized across the S.I.C.O.G. participating centers (see CONSORT diagram in Figure 1). Detailed patient characteristics are reported in Table 1. No significant imbalance of basic clinico-pathologic characteristics was observed between arms.

**Table 1:** Patients’ characteristics

Characteristics	FOLFIRI+B*12 (N=101)	FOLFIRI+B*6 (N=97)	*P* value$
Sex, female:male	34%:66%	42%:58%	0.2133
Age, median (range)	65years (34-81)	66years (26-80)	0.1531
Primary, right vs left colon	22% vs 78%	30% vs 70%	0.2163
Months since diagnosis, median(range)	2 (1-93)	2 (1-76)	0.4896
Grading, % of grade 1-2 vs grade 3	63% v 37%	74% v 26%	0.1848
Resection of the primary	77%	72%	0.4136
ECOG PS, 0:1	81%:19%	80%:20%	0.8901
Alkaline Phosphatase , % > ULN	9%	4%	0.1755
LDH, % > ULN	18%	12%	0.4545
WBC, median (range)	7 #/mm3(2.6-16.8)	6.6 #/mm3 (3.2-17.2)	0.1558
Neutrophils, median (range)	4.3 #/mm3 (1.7-16.7)	4.0 #/mm3 (1.4-13.5)	0.1098
Hemoglobin, median (range)	12.3 g/dL (8-16)	12.1 g/dL (8.8-16.1)	0.5318
Platelets, median (range)	254 #/mm3 (112-595)	260 #/mm3 (105-552)	0.9203
CEA, median (range)	24.4 mcg/mL (0.2-5518)	16.6 mcg/mL (0.1-5501)	0.6684
CA19.9, median (range)	35.7 UI/mL (0.1-44587)	31.9 UI/mL (0.6-203600)	0.7753
# of metastatic sites, % 1 vs ≥2	36% v 64%	40% v 60%	0.3131

Values refer to percentage of patients, unless otherwise specified. B: bevacizumab; ECOG PS: Eastern Cooperative Oncology Group Performance Status; ULN: upper limit of normal; LDH: lactate dehydrogenase; WBC: white blood cells.

$P values are calculated using chi-square test for categorical variables and t-test for continuous variables.

**Figure 2:**
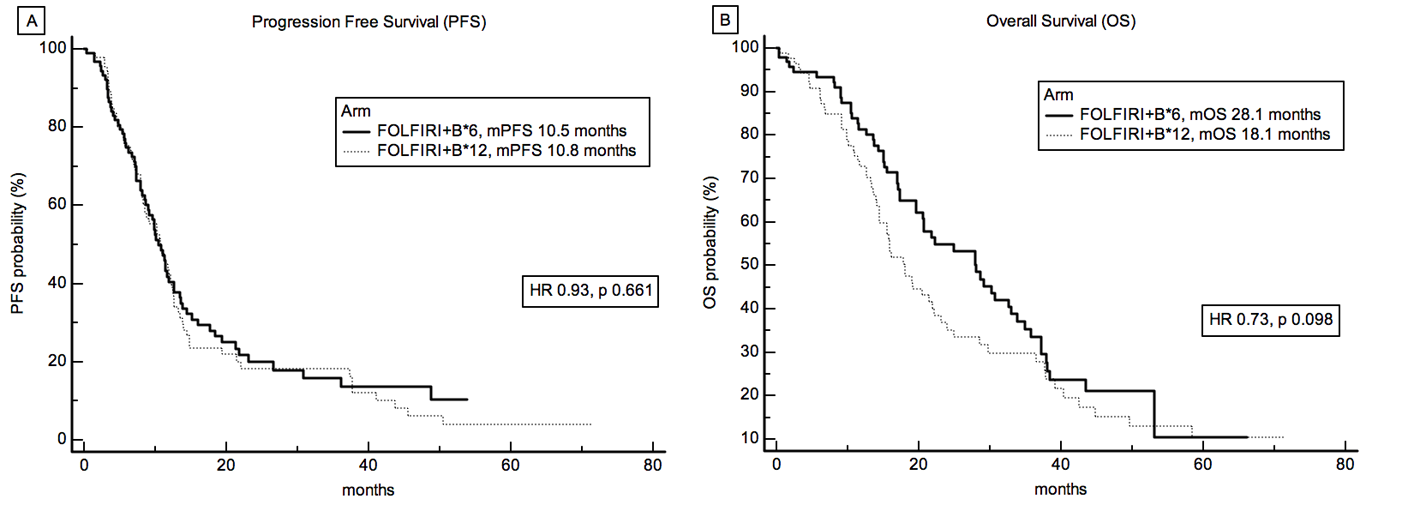
Survival analysis and treatment **A.** Progression Free Survival (PFS) and **B.** Overall Survival (OS) curves according to treatment arm. Median PFS was 10.8 and 10.5 months, and median OS was 18.1 and 28.1 months, in FOLFIRI+B*12 and FOLFIRI+B*6 arm, respectively.

After a median follow-up of 35.7 months for surviving patients, 136 patients had reached the progression endpoint, 118 patients had died. Median number of administered FOLFIRI+B was 12 (range 1-12) and 6 (range 1-6) for FOLFIRI+B*12 arm and FOLFIRI+B*6 arm, respectively. No significant difference was observed between the two arms for the primary endpoint of PFS (Figure 2A), with a median PFS of 10.8 and 10.5 months, in FOLFIRI+B*12 arm and FOLFIRI+B*6 arm, respectively, Hazard Ratio (HR) 0.9282 (95% CI 0.6640 to 1.2975), p 0.661. No difference of ORR was also detected, 41% *vs* 46%, respectively, chi-square *P* value = 0.4984. In terms of overall survival, although FOLFIRI+B*6 demonstrated a numerically superior median OS (18.1 and 28.1 months, for FOLFIRI+B*12 arm and FOLFIRI+B*6 arm respectively, HR 0.7402, 95%CI 0.5150 to 1.0638), this was not statistically significant, *P* = 0.0980 (Figure 2B).

**Figure 3:**
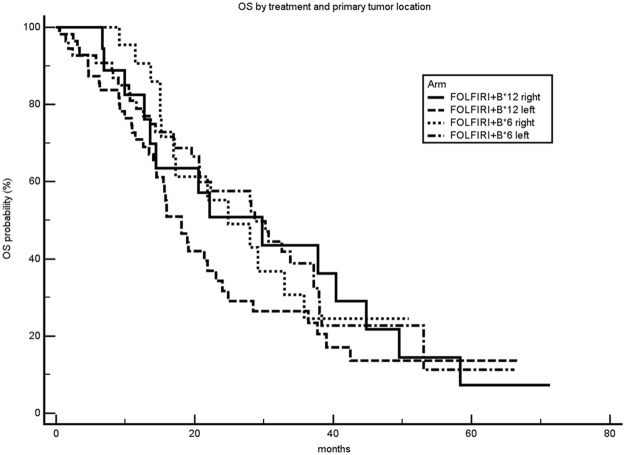
Overall survival curves according to treatment arm and primary tumor location mOS: median overall survival.

OS analysis of treatment arms was also performed by stratifying patients for primary tumor location. No significant difference between treatment arms was observed across different primary tumor locations (right *vs* left colon), Figure 3.

**Figure 4:**
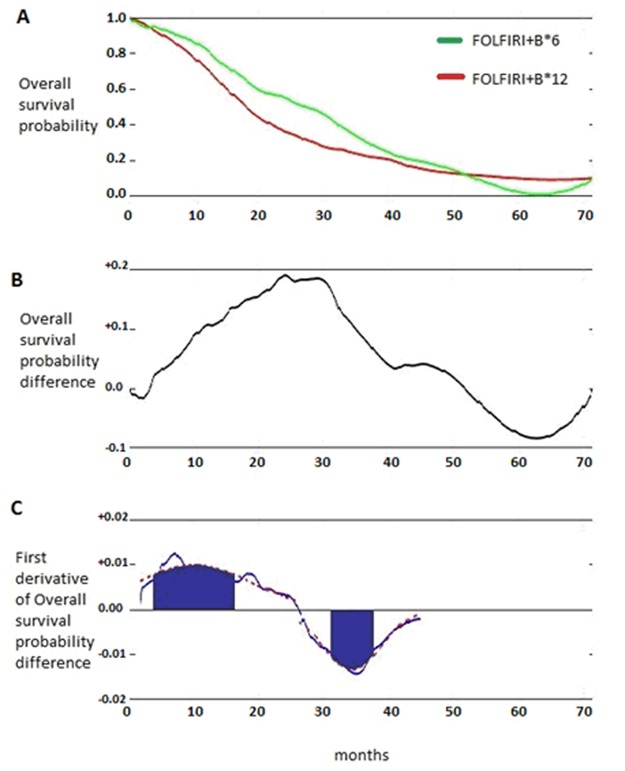
Death Pace Analysis of MARTHA trial **A.** Overall survival curves as in figure 2B; **B.** function of the survival probability difference between arms over the time; **C.** function of the first derivative of survival probability difference between arms over the time.

To identify the specific subset of patients benefiting in terms of OS from FOLFIRI+B*6 administration (shorter duration of Irinotecan treatment), a post-hoc Death Pace Analysis (DPA) was retrospectively carried out (Figure 4) as previously described [12, 13].

Briefly, DPA is based on the concept that the maximum treatment effect in terms of survival is observed in the time frame when the divergence between survival curves is generated and, when a ‘banana-shape’ of the curves is evident (see Figure 2B and 4A), the treatment benefit is lost when the curves converge. To capture the maximum treatment benefit, the first derivative of the difference between the survival probabilities is taken into consideration (Figure 4C). The first derivative represents, in this case, the rapidity with which a survival difference is being generated. It takes positive values when curves diverge, null values when survival curves run in parallel (i.e. the pace at which deaths occur is the same in the two treatment arms), and negative values when curves converge again.

The first derivative function helps more easily identify patients who effectively determine survival differences, since, as in the case of ‘banana-shaped’ curves, they are those who die in the inferior treatment arm when the positive first derivative ‘peak’ is generated and those who die in the superior treatment arm when the negative first derivative ‘peak’ takes place. Without these ‘peak’-generating patients the curves would be completely superimposable and of course no survival difference would be observed. It is possible to fit the first derivative function with two Gaussian Curves (one positive and one negative) providing the two peaks and relevant surrounding areas (median values and, arbitrarily chosen, one-third of their standard deviations). The Gaussian curves define the ‘time regions’ where truly survival-benefiting patients are ‘located’.

According to DPA, FOLFIRI+B*6-benefiting patients would be those dying between the 6^th^ and 14^th^ month in arm FOLFIRI+B*12 and those dying between the 32^nd^ and 36^th^ month in arm FOLFIRI+B*6 (Figure 4C). In the MARTHA trial, only 14% of patients (*n* = 27) of the whole population were DPA-defined FOLFIRI+B*6-benefiting patients.

A post-hoc univariate and multivariate logistic regression analysis (LRA) including baseline clinical and biochemical variables was then performed to identify distinctive characteristics of DPA-defined FOLFIRI+B*6-benefiting patients (Table 2). For the LRA, DPA-defined FOLFIRI+B*6-benefiting patients were coded as 1, and patients defined by DPA as not FOLFIRI+B*6-benefiting or with uncertain benefit (i.e. censored for overall survival before a sufficient follow-up time) were coded as 0. According to LRA, low haemoglobin level was the most characterizing feature of DPA-defined FOLFIRI+B*6-benefiting patients (independent predictor), with a 23% increase in the chance of detecting a FOLFIRI+B*6-benefiting patient for 1 gr/dL decrease in haemoglobin levels, Odds Ratio 0.7728 (95% CI 0.5992 to 0.9967), p 0.0471. A ROC (receiver operating characteristic) curve analysis was carried out to define the most discriminatory Hb cut-off value for the identification of FOLFIRI+B*6-benefiting patients. Hb value of 11.1 was demonstrated to be the best cut-off (Area under the ROC curve 0.634, Sensitivity 56%, Specificity 77%).

**Table 2:** Univariate and multivariate logistic regression analysis for predicting Death Pace Analysis-defined FOLFIRI+B*6-benefiting patients

*UNIVARIATE*						
**Variable**	**Coefficient**	**Std. Error**	**Wald**	**Odds ratio**	**95% CI**	***P***
**Hb (continuous)**	**-0.28939**	**0.12906**	**5.0274**	**0.7487**	**0.5814 to 0.9642**	**0.0249**
**Neutrophils (continuous)**	**0.16301**	**0.078995**	**4.2585**	**1.1771**	**1.0082 to 1.3742**	**0.0391**
Grading 1-2 vs 3	0.61291	0.36786	2.776	1.8458	0.8975 to 3.7959	0.0957
# of metastatic sites, 1 vs ≥2	0.34927	0.21209	2.7118	1.418	0.9357 to 2.1489	0.0996
WBCs (continuous)	0.11209	0.073299	2.3384	1.1186	0.9689 to 1.2914	0.1262
PLTs (continuous)	0.0029769	0.001967	2.2905	1.003	0.9991 to 1.0069	0.1302
Liver function tests, normal vs abnormal	1.23474	0.89316	1.9112	3.4375	0.5970 to 19.7930	0.1668
Sex, male vs female	0.6346	0.46612	1.8535	1.8863	0.7565 to 4.7030	0.1734
adjuvant chemotherpy, yes vs no	-0.71958	0.56889	1.5999	0.487	0.1597 to 1,4850	0.2059
Resection of the primary, yes vs no	-0.46082	0.44581	1.0684	0.6308	0.2633 to 1.5113	0.3013
primary tumor location, right vs left	0.41468	0.50124	0.6845	1.5139	0.5668 to 4.0435	0.4081
Time Dx to first-line (continuous)	-0.011158	0.016047	0.4835	0.9889	0.9583 to 1.0205	0.4868
CEA (continuous)	0.00012735	0.00024302	0.2746	1.0001	0.9997 to 1.0006	0.6003
LDH, normal vs abnormal	0.18529	0.39352	0.2217	1.2036	0.5565 to 2.6028	0.6378
CA19.9 (continuous)	-0.000010998	0.000030538	0.1297	1	0.9999 to 1.0000	0.7188
ALP, normal vs abnormal	0.14764	0.79944	0.0341	1.1591	0.2419 to 5.5542	0.8535
Age (continuous)	0.0029536	0.020514	0.02073	1.003	0.9634 to 1.0441	0.8855
ECOG PS, 0 vs 1	-0.050858	0.53198	0.00914	0.9504	0.3350 to 2.6961	0.9238
***MULTIVARIATE***						
**Variable**	**Coefficient**	**Std. Error**	**Wald**	**Odds ratio**	**95% CI**	***P***
**Hb (continuous)**	**-0.25772**	**0.12979**	**3.9428**	**0.7728**	**0.5992 to 0.9967**	**0.0471**
Neutrophils (continuous)	0.13862	0.080305	2.9797	1.1487	0.9814 to 1.3445	0.0843

Only variables found to be significant at univariate analysis (*p* < 0.05) were analysed in the multivariate analysis. Hb: haemoglobin; WBCs: white blood cells; PLTs: platelets, Dx: diagnosis; LDH: lactate dehydrogenase; ALP: alkaline phosphatase; ECOG PS: Eastern Cooperative Oncology Group Performance Status

As many as 56% of DPA-defined FOLFIRI+B*6-benefiting patients had Hb ≤ 11.1 as compared to 23% of non- FOLFIRI+B*6-benefiting patients, chi square *P* value = 0.0005.

**Figure 5:**
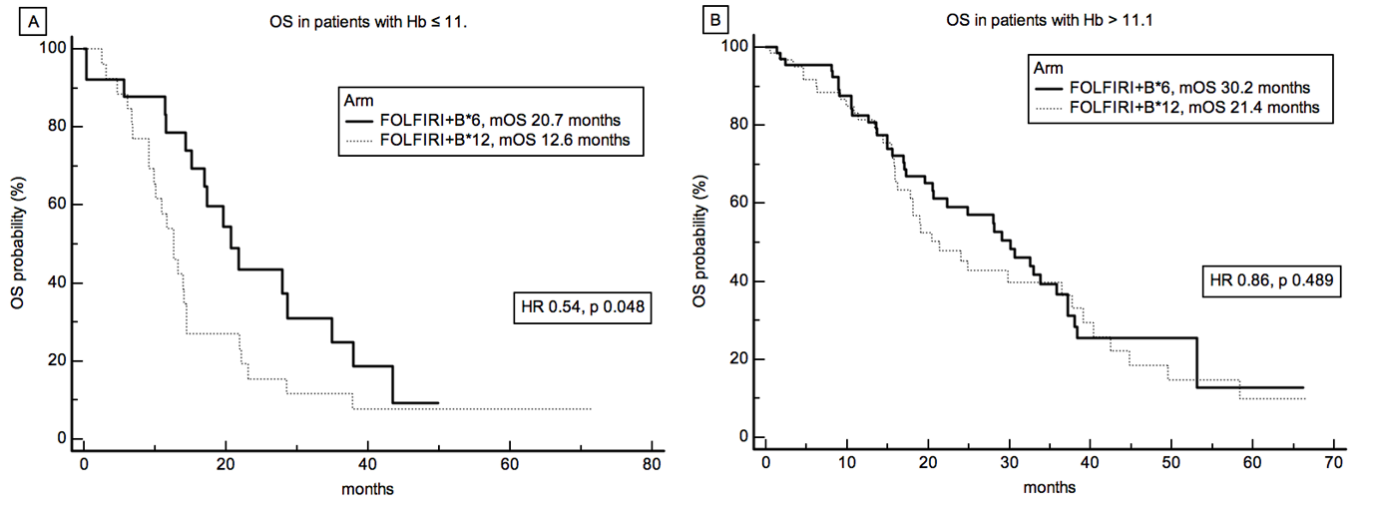
Survival Analysis and Hemoglobin levels Overall survival by treatment arm of patients stratified according to Hb ≤ 11.1(**A**) or Hb >11.1 (**B**).

In confirmation of the above, patients with low (≤ 11.1) and high (>11.1) baseline Hb levels showed distinct overall survival curves (Figure 5). Indeed, a statistically significant improvement in survival was observed for patients treated with short duration of irinotecan treatment in the low Hb subgroup, mOS 12.6 months *vs* 20.7 months, for FOLFIRI+B*12 and FOLFIRI+B*6, respectively, HR 0.54, *P* = 0.048 (Figure 5A). Interestingly, a cox proportional hazard regression analysis for overall survival with interaction test did not demonstrate any significant interaction between treatment and Hb levels dichotomized as per the median value of 12.3 g/dL (data not shown).

### Toxicity

The ‘per protocol’ population was assessable for safety. Toxicity data were recorded prospectively according to CTCAE v 4.0. To underlie substantial safety differences between FOLFIRI+B*6 and FOLFIRI+B*12 arm, incidence of major grade 3-4 toxicities are reported in Table 3.

**Table 3:** Frequency of major grade 3-4 toxicities by treatment arm

Toxicity	FOLFIRI+B*12, % of patients with grade 3-4	FOLFIRI+B*6, % of patients with grade 3-4	*P* value
Neutropenia	20%	9%	0.04
Thrombocytopenia	2%	0%	0.14
Nausea/vomit	5%	3%	0.64
Diarrhea	12%	2%	0.01
Hypertension	0%	1%	0.33

Percentage of patients experiencing specific grade 3-4 toxicities during the entire course of treatment are reported.

No unexpected toxicity issues were observed, however significant less grade 3-4 neutropenia and diarrhea was observed for FOLFIRI+B*6 treated patients (9% and 2%, respectively) as compared to FOLFIRI+B*12 treated patients (20% and 12%, respectively), chi-square p values 0.04 and 0.01, respectively.

## DISCUSSION

In this randomized multicentre phase III trial, the MARTHA (Maintenance and Reduction Chemotherapy With Avastin in Metastatic Colon Cancer) trial, a clinical study designed to compare 12 cycles *vs* 6 cycles of first-line FOLFIRI+bevacizumab, with both arms then followed by de-intensified maintenance therapies, we were not able to demonstrate the superiority of a standard strategy of FOLFIRI+Bevacizumab given for approximately six months as compared to a short duration, three months, full treatment schedule.

Primary tumor sidedness has lately gained great attention as important prognostic (and possibly predictive) factor and seems to reflect substantial differences from both a molecular and microenvironmental viewpoint [14]. Dejea et al, have recently demonstrated that certain microbiotic profiles are associated to specific colon cancer location and takes part in colon carcinogenesis [15]. The microbiotic profile in right *vs* left colon is substantially distinct and may partly account for the prognostic/predictive value of tumor sidedness, however the precise underlying biological mechanisms still remain largely unknown. This clinical effect seems to be particularly evident in RAS wild type tumours, and for patients treated with anti-EGFR agents, while the survival differences by primary tumour side are less clear for anti-VEGF agent-treated patients [16]. Although as a post-hoc analysis, the effect of primary tumor location on treatment efficacy was investigated in the MARTHA trial. No interaction was demonstrated between treatment and tumor sidedness, and no prognostic influence was also observed. The MARTHA trial was conducted in a pre-RAS/BRAF era and data on RAS/BRAF mutational status were largely lacking, thus we were not able to analyze tumor sidedness effect in the wild type patient subpopulation. Furthermore, the trial was designed to provide the anti-VEGF agent bevacizumab to both treatment arms. It is possible that the lack of prognostic impact of primary tumor location in our study (p 0.3581) could be attributable to an imbalance of RAS mutated tumors in left colon cancers or to the levelling of clinical outcome obtained with the use of bevacizuamb in all patients.

In the primary analysis, a non-statistically significant longer survival was associated with FOLFIRI+B*6. We used for the first time a novel mathematical analysis, the ‘death pace analysis’ (DPA), to unravel possible clinical or biochemical features able to identify patients most benefiting from FOLFIRI+B*6 and make the difference between arms statistically significant.

The retrospective DPA helped determine that in patients with low baseline haemoglobin (Hb ≤ 11.1) a short duration of irinotecan therapy might be more beneficial as compared to prolonged irinotecan administration.

We acknowledge that the trial was designed to demonstrate the superiority of six months over three months FOLFIRI+Bevacizumab, and the possible superiority of three months therapy in specific subpopulations, or even its non-inferiority in the general population, would require e different statistical planning and sample size calculation. However, if this finding were confirmed with an adequate sample size, the reduced duration of full treatment would have a primary positive impact in terms of safety, as seen in Table 3.

The explanation of why prolonged exposition to irinotecan would have detrimental effects in patients with low Hb levels remains unknown. It is possible that patients with low haemoglobin levels may have tumors at increased risk of developing chemo-resistant and aggressive sub-clones with the prolonged use of irinotecan. It has been recently reported that prolonged exposition to irinotecan determined, in certain tumor cell lines, the up-regulation of γ-H2AX and phospho-Chk2, two DNA damage signal proteins able to enhance DNA repair thus reducing chemotherapy activity. High γ-H2AX and phospho-Chk2 confer an aggressive phenotype and cross-resistance to other chemotherapeutics, such as fluorouracil [17]. It has been demonstrated, that hypoxic environment such as that determined by low haemoglobin levels, may particularly favour this type of resistence [18].

Another possible explanation could be that low Hb patients represent a frail population at risk of clinical deterioration, and hence of worse outcome, for prolonged use of full dose chemotherapy. On that respect, it would have been informative to analyze data on second line treatment and see whether low Hb patients in the FOLFIRI+B*12 arm were less likely to receive second line chemotherapy with resulting negative impact on survival. Unfortunately, data on second line therapy were not available in the MARTHA trial.

Overall, results of the MARTHA trial were comparable to those of larger maintenance trials. Median PFS and OS were reported to be 8.5 and 21.6 months, respectively, in the capecitabine/bevacizumab maintenance arm of the CAIRO3 study in a recently updated analysis [19]. These survival results were very close to the 10.1 and 25.2 months of median PFS and OS, respectively, observed in our study in the whole population (data not shown). Similar results were also obtained in other three studies, AIO0207, SAKK41/06 and NCT00623805, evaluating bevacizumab-based maintenance regimens [20–22].

In conclusion we could not demonstrate a superiority of standard duration FOLFIRI+bevacizumab over short-term duration of irinotecan administration. On the contrary, our data demonstrate a detrimental effect of prolonged irinotecan use in patients with low haemoglobin level. In this particular subset of patients for whom a standard FOLFIRI+bevacizumab is planned, en effort to increase Hb concentration should be taken into consideration and the use of supportive drugs to improve red cell line myelopoiesis, such as erythropoietin, would be recommended.

## MATERIALS AND METHODS

### Patient enrollment

Chemotherapy-naïve patients with histologically proven colorectal cancer and measurable metastatic disease were deemed eligible. Patients were randomized in 23 participating centers within the S.I.C.O.G. (Southern Italy Cooperative Oncology Group) in a 1:1 ratio to one of the following two arms: FOLFIRI+B*12 or FOLFIRI+B*6 (see below for the schedules).

Inclusion criteria were: Age > 18 years, Adjuvant treatment ended ≥ 6 months before study entry, no prior exposure to irinotecan and/or bevacizumab, no prior exposure to cytotoxic drugs for the metastatic disease, ECOG Performance Status 0-1, adequate hematologic, coagulative, renal and hepatic functions, no evidence of proteinuria. Exclusion criteria were: untreated brain metastases or spinal cord compression, non-healing wound or bone fracture, evidence of bleeding diathesis or coagulopathy, uncontrolled hypertension, clinically significant cardiovascular disease, other co-existing malignancies or malignancies diagnosed within the last 5 years with the exception of basal and squamous cell carcinoma or cervical cancer in situ, major surgical procedure or significant traumatic injury within 28 days prior to study treatment start, pregnant or lactating women, any psychological or social condition that may interfere with the participation into the study or the evaluation of study results

### Trial design and conduction

The protocol was approved by research and ethics committees at each participating center and complied with Good Clinical Practice guidelines and the principles of the Declaration of Helsinki, and local laws. All patients had to sign written informed consent for the study. Patients in FOLFIRI+B*12 arm received: irinotecan 180 mg/m2 90 min. i.v. infusion, levo-folinic acid 200 mg/m2 120 min. i.v. infusion, 5-fluorouracil 400 mg/m2 i.v. bolus, Bevacizumab 5 mg/kg 30 to 90 min. i.v. infusion and 2,400 mg/m2 continuous i.v. infusion over 46 hours; all on day 1 every 2 weeks for 12 cycles (6 months); afterwards, Bevacizumab 7.5 mg/kg i.v. every 3 weeks up to 1 year was delivered (i.e. further 6 months of therapy). Patients in FOLFIRI+B*6 arm received the same regimen as FOLFIRI+B*12 for 6 cycles (3 months), followed by capecitabine 1250 mg/sqm orally twice daily, 12 hours apart (2,500 mg/sqm daily dose) from day 1 to day 14 plus Bevacizumab 7.5 mg/kg i.v. on day 1, every 3 weeks for 4 cycles (further 3 months) and then monotherapy with Bevacizumab 7.5 mg/kg i.v. every 3 weeks up to 1 year (i.e. further 6 months of therapy).

Standard hematological and biochemical tests were performed before each cycles.

Treatment activity was assessed every three months with a contrast enhanced thorax/abdomen/pelvis CT scan (or with plain thorax CT scan plus abdomen-pelvis MRI, if iodinated contrast was not indicated).

### Statistical considerations

Primary endopoint was progression free survival (PFS) and was set to demonstrate the superiority of FOLFIRI+B*12 to FOLFIRI+B*6 (EUDRACT Number 2008-004890-17). For a 80% power and a two-tail 5% alpha error, 186 patients (93 per arm) were required to demonstrate an increase of 6-month-PFS rate from 50% to 70%.

Secondary endpoints were overall survival (OS), overall radiologic response rate (ORR as assessed according to RECIST 1.1 criteria) and toxicity, graded as per Common Terminology Criteria for Adverse Events (CTCAE) version 4.0.

Progression-free survival was defined as the interval time between study enrolment and progression according to RECIST criteria or death from any cause. Overall survival was defined as the interval time between study enrolment and death from any cause.

Survival curves and survival differences between arms or patient subgroups were analysed using the Kaplan-Meier method with the log-rank test. A ‘death pace analysis’ (DPA) was performed to identify patients benefiting more from a specific treatment arm [12, 13] and a logistic regression analysis (LRA), including common clinical and biochemical baseline variables, was used to define features characterizing patients identified by DPA. ROC analysis was applied when discriminatory cut-off values of continuous variables had to be determined. Differences between patient subgroups of categorical and continuous variables were assessed using chi-square and Mann-Whitney test, respectively. All statistical tests except DPA were performed using MedCalc for Windows, version 15.0 (MedCalc Software, Ostend, Belgium). DPA was performed using MATLAB (MATLAB 6.1, The MathWorks Inc., Natick, MA, 2000).

We thank Dr. Christine Tracey for revising the English and contributing to the clarity of the information provided in the present article. We thank dr Cristiano Serci for his precious work of data management
